# Chlorothalonil exposure induces “liver-gut axis” disorder in mice: Liver-gut axis disorder in mice

**DOI:** 10.3724/abbs.2022078

**Published:** 2022-07-12

**Authors:** Huaping Tao, Zhiwei Bao, Yuanxiang Jin

**Affiliations:** 1 College of Biotechnology and Bioengineering ZhejiangUniversity of Technology Hangzhou 310032 China; 2 Institute of Life Sciences Key Laboratory of Organ Development and Regeneration of Zhejiang Province College of Life and Environmental Sciences Hangzhou Normal University Hangzhou 311121 China

Chlorothalonil (CTL) is a broad spectrum, non-systemic, organochlorine fungicide, widely used in agriculture, silviculture, urban settings, and industrial antifouling. It is found in various environmental media, such as surface water, soil, and air, and even exceeded Maximum residual limit (MRL) in food chain [ 1, 2] . Previous studies revealed that CTL is highly toxic to aquatic organisms and amphibian, especially in the early stage of development [3]. Under experimental conditions, it may also have toxic effects on rodents, mammalian cells, and other non target organisms. In humans, the main exposure modes of CTL were contacting to the residues in the work/life place or intaking the contaminated dietary, which may cause contact allergic dermatitis, occupational asthma, gastrointestinal problems and other symptoms [4]. However, the effects of CTL on the “liver-gut axis” have not been reported yet.

In this study, after one week of adaptation to the environment, six-week-old male ICR mice (China National Laboratory Animal Research Center, Shanghai, China) with almost the same weights were weighed and randomly assigned into 4 groups ( *n*=8). The control group and 3 experimental groups were orally administered with 0, 200, 400 and 800 μg/L CTL respectively in drinking water every day for 9 weeks. Then after 12 h of fasting, all mice were anesthetized by ether, and the liver, ileum and colon were collected quickly and frozen in liquid nitrogen, then kept at −80 ^o^C until further analysis. All animal experiments were performed in accordance with the Guiding Principles in the Use of Animals in Toxicology.

During the 9-week exposure, no significant change in body weight was observed between any CTL-treated group and the control group. The hepatic glucose (GLU) level, lipoprotein lipase (LPL) activity, glutamic pyruvic transaminase (GPT) activity, and glutamic oxalocetic transaminase (GOT) activity changed significantly after 9 weeks of CTL exposure, indicating that the liver function was influenced. Other indicators did not change significantly ( Supplementary Table S1).

The expressions of genes related to glycolipid metabolism were measured by qRT-PCR using ChamQ Universal SYBR qPCR Master Mix (Vazyme, Beijing, China) and primers listed in Supplementary Table S2. As shown in Figure 1A, CTL exposure notably down-regulated the expressions of genes related to glycolipid metabolism. For example, pruvate kinase (PK) and carbohydrate regulatory element binding protein (Chrebp), which are responsible for glycolysis, showed a down-regulated trend in CTL-treated groups when compared with the control group. Their down-regulation could raise the GLU storage and reduce hepatic pyruvate level in the liver. However the change of transcriptional level of glucokinase (GK) is not clear. The genes involved in fatty acid β-oxidation, such as peroxisome proliferator-activated receptor α (PPAR-α), Cpt1a and ACOX, all showed a down-regulated trend, especially in the CTL-800 group ( Figure 1B). CTL exposure also reduced the expressions of genes participated in fatty acid synthesis and transportin the liver, such as PPAR-γ, citrate lyase (Acl), stearoyl COA desaturase 1 (Scd1) and fatty acid binding protein (Fabp1) ( Figure 1C,D). The mRNA levels of genes related to TG synthesis, including diacylglycerol acyltransferase 2 ( *Dgat2*) and glyceraldehyde 3-phosphate acyltransferase ( *Gpat*) were also declined in the CTL-800group ( Figure 1E). Maybe it is a stress response to hyperlipidemia in the liver. These results suggested that CTL exposure reduced the ability of glycolipid metabolism in the liver through downregulating thetranscriptional levels of key genes.

**Figure 1:**
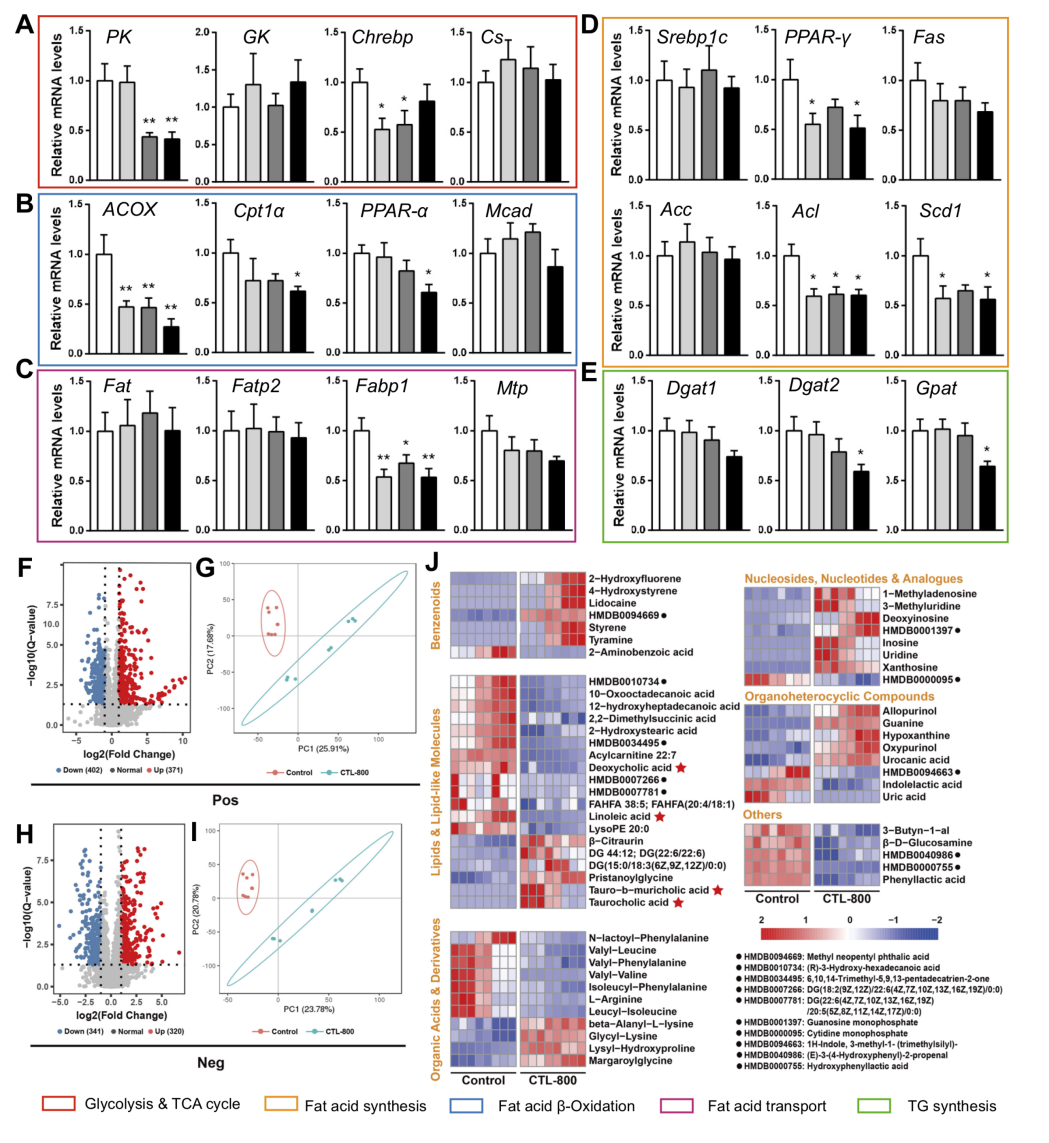
Figure 1 Effects of CTL exposure on the transcription of genes related to metabolism in the liver of mice (A−E) CTL exposure induced transcriptional responses of genes related to glycolysis, β-oxidation, fatty acid synthesis, TG transportation and synthesis in the liver. The values are presented as the mean±SEM. * P<0.05, ** P<0.01. (F−J) Hepatic metabolites change after CTL-800 exposure. Fold changes of metabolites levels from the control and CTL-800 groups; (F) In pos-model; (H) In neg-model. OPLS-DA score plots of liver samples from the control and CTL-800 groups; (G) In pos-model; (I) In neg-model. Supervised analysis techniques of partial least-squares discriminant analysis (PLS-DA) were used. The quality of the model was described by the cross-validation parameters R2=(0.0, 0.86), Q2=(0.0, −0.686) in pos-model and R2=(0.0, 0.8707), Q2=(0.0, −0.693) in neg-model; (J) Heat map illustrated the difference of hepatic metabolites between the control and CTL-800 groups. Samples are colored by treatment to show the grouping. The amount of variation attributable to each principal component is indicated in the axis titles. The row represents the metabolites, and the column represents the individual samples. Red bands indicate up-regulated metabolites, and blue bands indicate down-regulated metabolites in the two groups. The deeper the color, the greater the difference in metabolites.

We further analyzed hepatic metabolites by liquid chromatography tandemmass spectrometry (LC-MS/MS) between the control group and CTL-800 group. Liver tissue extraction was performed as previously reported [5]. Through principle component analysis (PCA), we found that the patterns of metabolites were significantly different from both positive and negative between the control and CTL-800 group ( Figure 1G,I). Among of the high-quality feature, a total of 371 metabolites were increased and 402 metabolites were decreased in the positive-model ( Figure 1F). Correspondingly, a total of 320 metabolites were increased and 341 metabolites were decreased in negative-model ( Figure 1H). We screened the metabolites (|log2(Fold Change)|≥1, VIP ≥1, Q value≤0.05) obtained from MS2 according to the HMDB database and observed significant changes in benzenoids, organic acids and derivatives, organ oheterocyclic compounds, lipids and lipid-like molecules, organic oxygen compounds, organooxygen compounds, nucleosides, nucleotides, and analogues between the control group and CTL-800 group ( Figure 1J). Among these, the contents of deoxycholic acid, linoleic acid, tauro-b-muricholic acid and taurocholic acid which are related to bile acids (BAs) metabolism were found to have different degrees of changes after exposure to 800 μg/L CTL, indicating that BA metabolism was also disrupted.

We further detected the transcriptional levels of genes responsible for BAs synthesis, transportation and the signal molecular of BAs hepatointestinal circulatory and found that the expression of cyp7a1, a rate-limiting enzyme of BAs synthesis, was significantly suppressed at the transcriptional level in the CTL-800 treated group ( Figure 2A). MRP3, which is related to BAs secretion into systemic circulation, was significantly up-regulated in all CTL treatment groups.

**Figure 2:**
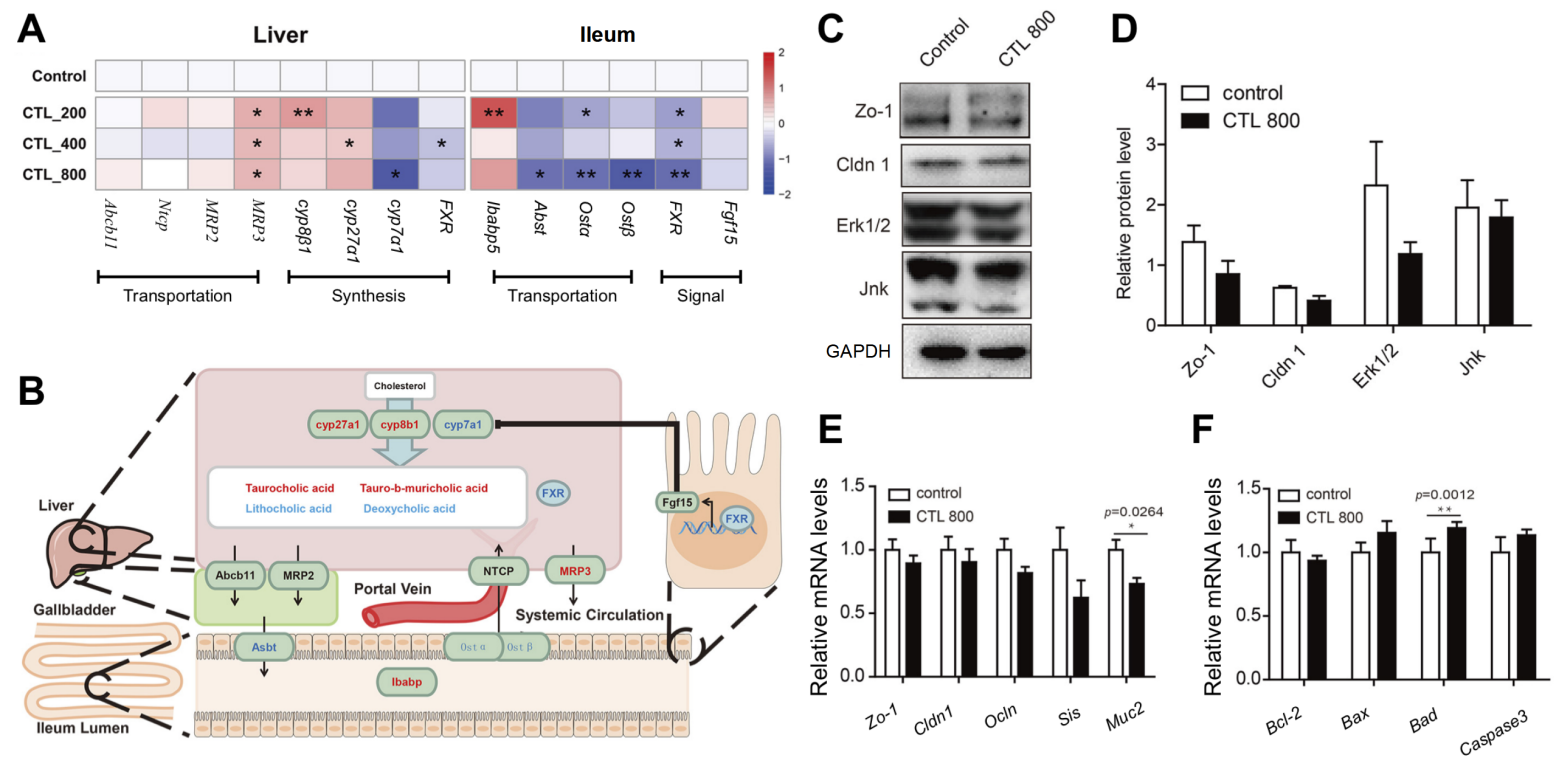
Figure 2 Effects of CTL exposure on BAs metabolism and intestinal barrier integrity in mice (A) Gene expression change involved in BAs intra-hepatic synthesis, secretion in liver, reabsorption in ileum and negative feedback signal. The deeper the color, the greater the difference in metabolites. (B) Diagram of “liver-gut axis” circulation metabolism of BAs. Red bands indicate up-regulated genes, and blue bands indicate down-regulated genes. (C) Western blot analysis for tight junction proteins (Zo-1 and Cldn 1) and MAPK signaling pathway proteins (ERK1/2 and JNK). (D) Relative expression levels of MAPK proteins are standardized with respect GAPDH protein. (E,F) qRT-PCR for mRNA levels of genes related to apoptosis ( Bcl-2, Bax, Bad, and Caspase 3), tight junction ( Zo-1, Cldn 1 and Ocln) and intestinal function ( Sis and Muc 2). All values are presented as the mean±SEM. * P<0.05, ** P<0.01 compared with control group.

Meanwhile, genes regulating BAs re-absorption in the ileum were equally perturbed by CTL exposure ( Figure 2A,B). Abst, Ostα and Ostβ were significantly down-regulated in high dosage group, and both Ostα and Ostβ were decreased even in low dosage group. Farnesoid X receptor (FXR) is a member of the nuclear receptor family regulating the expression of Fgf15, which regulates the negative feedback of BA synthesis [6]. It was decreased significantly in all CTL exposure groups in the ileum and in the liver of the CTL-400 group ( Figure 2A). In addition, all the possible roles of these genes in the pathways in liver-intestine circulation metabolism of BAs are shown in Figure 2A.

The live and gut have the same embryological origin. They have a natural and extensive connection in structure and function. The intestinal blood reflux forms the portal vein system into the liver. Intestinal toxins require the liver to rely on its innate immune system to play a defensive role. The liver regulates metabolism and immune response, and affects intestinal function through bile secretion and enterohepatic circulation. Liver disease can lead to the weakening of immune defense mechanism of intestinal barrier and the damage of tight junction of epithelial cells [1]. Their pathophysiological relationship is expressed as “liver-gut axis”. The imbalance of BAs metabolism will also affect the physiological function between liver and intestine [7]. Our previous studies confirmed that CTL exposure will increase intestinal cell apoptosis and destroy the integrity of intestinal barrier on Caco-2 monolayer model [ 8, 9] .

Based on this finding, we detected the related indexes of intestinal barrier integrity by western blot analysis of the colon samples collected as previously described [9] using antibodies listed in Supplementary Table S3. We found that the expressions of tight junctions (TJs) proteins Zo-1 and *Cldn 1* had downward trends, and the expressions of Erk1/2 and Jnk, two key members in MAPK signaling pathway in the regulation of intestinal epithelium barrier stability, were also declined ( Figure 2C,D). Quantitative PCR revealed that the expressions of TJ genes, including *Zo-1*, *Cldn* 1 and *Ocln*, had down-regulated trend, which is the same as that of the *Sis* (Brush border marker) gene, and the change of *Muc2* is significant ( Figure 2E). At the same, the expressions of apoptosis-related genes, *Bax* and *Caspase 3* showed up-regulated trend, the change of *Bad* is significant, andexpression of *Bcl-2* was accordingly decreased, though it is not significant ( Figure 2F).

In summary, in this study we observed that sub-chronic exposure to CTL disturbed “liver-gut axis” metabolism, mainly including glucose and lipid metabolism and intestinal barrier integrity, at the transcription and protein expression level. In addition, the metabolism of BAs which are one of the bridges connecting the liver and gut is also influenced. Based on the results, we speculate that CTL may have toxic effects on liver metabolism through disturbing BAs metabolism. With regard to the “liver-gut axis”, the damage of the intestinal barrier may also be involved in the functional abnormalities of liver metabolism.

## Supplementary Data

Supplementary data is available at *Acta Biochimica et Biophysica Sinica* online.
